# Emerging Roles of T Helper Cells in Non-Infectious Neuroinflammation: Savior or Sinner

**DOI:** 10.3389/fimmu.2022.872167

**Published:** 2022-06-30

**Authors:** Wenbin Liu, Meiyang Fan, Wen Lu, Wenhua Zhu, Liesu Meng, Shemin Lu

**Affiliations:** ^1^ Institute of Molecular and Translational Medicine, and Department of Biochemistry and Molecular Biology, School of Basic Medical Sciences, Xi’an Jiaotong University Health Science Center, Xi’an, China; ^2^ Department of Neurosurgery, The First Affiliated Hospital of Xi’an Jiaotong University, Xi’an, China; ^3^ Department of Psychiatry, The First Affiliated Hospital of Xi’an Jiaotong University, Xi’an, China; ^4^ National Joint Engineering Research Center of Biodiagnostics and Biotherapy, Second Affiliated Hospital, Xi’an Jiaotong University, Xi’an, China; ^5^ Key Laboratory of Environment and Genes Related to Diseases (Xi’an Jiaotong University), Ministry of Education, Xi’an, China

**Keywords:** Th cells, neuroinflammation, multiple sclerosis, alzheimer’s disease, parkinson’s disease, epilepsy, traumatic brain injury (craniocerebral trauma), mental disorders

## Abstract

CD4^+^ T cells, also known as T helper (Th) cells, contribute to the adaptive immunity both in the periphery and in the central nervous system (CNS). At least seven subsets of Th cells along with their signature cytokines have been identified nowadays. Neuroinflammation denotes the brain’s immune response to inflammatory conditions. In recent years, various CNS disorders have been related to the dysregulation of adaptive immunity, especially the process concerning Th cells and their cytokines. However, as the functions of Th cells are being discovered, it’s also found that their roles in different neuroinflammatory conditions, or even the participation of a specific Th subset in one CNS disorder may differ, and sometimes contrast. Based on those recent and contradictory evidence, the conflicting roles of Th cells in multiple sclerosis, Alzheimer’s disease, Parkinson’s disease, epilepsy, traumatic brain injury as well as some typical mental disorders will be reviewed herein. Research progress, limitations and novel approaches concerning different neuroinflammatory conditions will also be mentioned and compared.

## Introduction

As proposed by Nobel Laureate Sir Perter Medawar, the central nervous system (CNS) is an immune-privileged site with tightly regulated immune responses. Dysregulated or imbalanced immune functions in the CNS could incur severe pathogenesis or complications of both neurological and psychological diseases. By the convenience of recent developments in neuroscience and immunology, accumulating evidence has emphasized the role of adaptive immunity in CNS diseases of immunological etiologies (e.g., multiple sclerosis), and also revealed the involvement or possible dominance of immunocytes and their cellular products in those CNS disorders that were once considered immune-unrelated.

Upon activation, naïve CD4^+^ T cells can differentiate into several types of professional cytokine-producing T helper (Th) cells, which contribute to host defense against pathogens. However, under certain circumstances they could also elicit tissue damage which further leads to chronic inflammatory disorders. Although proved present within the CNS in normal physiological conditions, Th cells are actually able to infiltrate and abound in the ‘immune-privileged site’ under various pathological conditions, including autoimmune CNS disorders, epilepsies, neurodegenerative disorders, traumatic brain injuries, mental disorders, and admittedly, many other CNS disorders. As their roles emerge, it’s also found that functions of one specific Th cell subset may vary regarding different CNS diseases, or even not remain constant in the same pathological condition. Thusly, a question arises: whether Th cells are saviors or sinners when the mysterious CNS is confronting pathological threats. Based on this dichotomy of “savior or sinner”, the participation of Th cells and their cytokines in several major CNS disorders will be reviewed with an emphasis on those controversial and recent discoveries. It should be noted that immunity against CNS malignancy or neoplasm will not be discussed herein.

## Th Cells: The Basics

### Subsets and Functions of Th Cells

Up to now, there are at least seven major Th cell subsets that are characterized by their lineage-defining cytokines including Th1, Th2, Th17, Th9, Th22, follicular T helper (Tfh) and regulatory T (Treg) cells, and these cells have distinct functions in immune responses (1–3). Th1 cells were first discovered in the late 1980s (4, 5). They are primarily responsible for defending against intracellular infections as well as the development of organ-specific autoimmunity. Th1 cells activate macrophages by producing interferon-γ (IFN-γ) and promoting the generation of opsonizing antibodies (Abs), which are considered as their major functions (6, 7). Th2 cells cause B-cell immunoglobulin (Ig) flipping toward IgG1 and IgE by releasing interleukin-4 (IL-4) (8), attract eosinophils by producing IL-5 (9) and also increase smooth muscle cell movement and mucus generation by epithelial cells through producing IL-13 (10–12). Th2 cells can also lead to macrophage activation by producing IL-4 and IL-13 (13). Th17 cells were discovered in 2005 (14), and they have a critical role in defending against extracellular pathogens like bacteria and fungi (15), as well as contributing to immunopathology in autoimmune disorders. Th17 cells secrete various cytokines including IL-17A, IL-17F, IL-21 and IL-22 (16). By increasing the expression of inflammatory cytokines and chemokines, as well as encouraging neutrophil recruitment in inflammatory regions, IL-17A and IL-17F act on multiple cell types, including macrophages, epithelial cells, and endothelial cells (17, 18). The successful amplification of Th17 cell population depends on the positive-feedback loop established by IL-21 (19). IL-22 is a vital cytokine for inducing the secretion of antimicrobial substances, as well as proinflammatory cytokines and chemokines, which are released by cells at mucosal barriers (20).

Sakaguchi et al. discovered in 1995 a CD4^+^CD25^+^ T cell fraction capable of suppressing effector T cells and maintaining immune tolerance (21), which is denoted as Treg cells. Two types of Treg cells have been identified. Natural Treg cells (nTreg), expressing the transcription factor forkhead box P3 (FoxP3), grow in the thymus in response to self-antigen recognition (22). The other type is named as inducible Treg cells (iTreg), which develop from naïve CD4^+^ T cells at certain TCR-stimulating circumstances in a specific cytokine milieu. FoxP3^+^ Treg cells, IL-10-producing type 1 regulatory (Tr1) T cells, as well as transforming growth factor-β (TGF-β)-producing Th3 cells, are all considered as subsets of iTreg cells (23). Treg cells govern the differentiation and functioning of effector T cells, thereby maintaining immunological tolerance and controlling the intensity of immune reaction (3, 18, 24, 25).

Early research from the 1990s suggested that the generation of IL-9 was primarily associated with Th2 cells (22). However, IL-4, in combination with TGF-β and IL-2, has been found to drive naïve CD4^+^ T cells to generate IL-9 *in vitro*, but no other Th2 signature cytokines (26). In 2008, the existence of a CD4^+^ T cell population that produces predominantly IL-9 *in vivo* (referred to as Th9 cells) was confirmed, and thereafter Th9 cells commenced to be linked to antitumor immunity, allergies, as well as autoimmune disorders (27). A distinct Th cell subgroup that secretes IL-22, known as Th22, was discovered in 2000 (28). Several other cell types, including Th17 cells, natural killer (NK) cells (29, 30), and macrophages, are also biological sources of IL-22 (31). IL-22 mainly act on non-hematopoietic cells (e.g., epithelial cells) to enhance epithelial barrier activities and promote the regeneration and proliferation of epithelial cells (32).

During immune responses, CD4^+^ T cells also play an important role by assisting B cells in producing antibodies and Ig class switching. Tfh cells, known as the CD4^+^ Th cells present in the B-cell follicle, have been proven crucial to these responses (24). Tfh cells are distinguished from Th1, Th2, Th17 as well as Treg cells, and are regarded as another Th cell subset (33). Tfh cells are divided into at least two categories, one of which produces IFN-γ and another produce IL-4. Truly, Tfh cells make up the majority of Th cells that produce IL-4 in the organism (34). Tfh cells that produce IL-4, unlike typical Th2 cells, do not express IL-13 (35). It’s worth noting that Tfh cells are a major source of memory Th cells, which are able to trans-differentiate into traditional Th effector cells upon reactivation (36).

Rather recently, a distinct granulocyte-macrophage colony-stimulating factor (GM-CSF)^+^ Th cell subset was identified and named ThGM cells, which are primed to acquire a Th1 phenotype and cause neuroinflammation. TNF, IL-2, IL-3, and CCL20 comprise the major secretion profile of ThGM cells, and simultaneously they lack the production of Th lineage–specific cytokines and transcription factors (37).

### Differentiation of Th Cells

**Figure 1:**
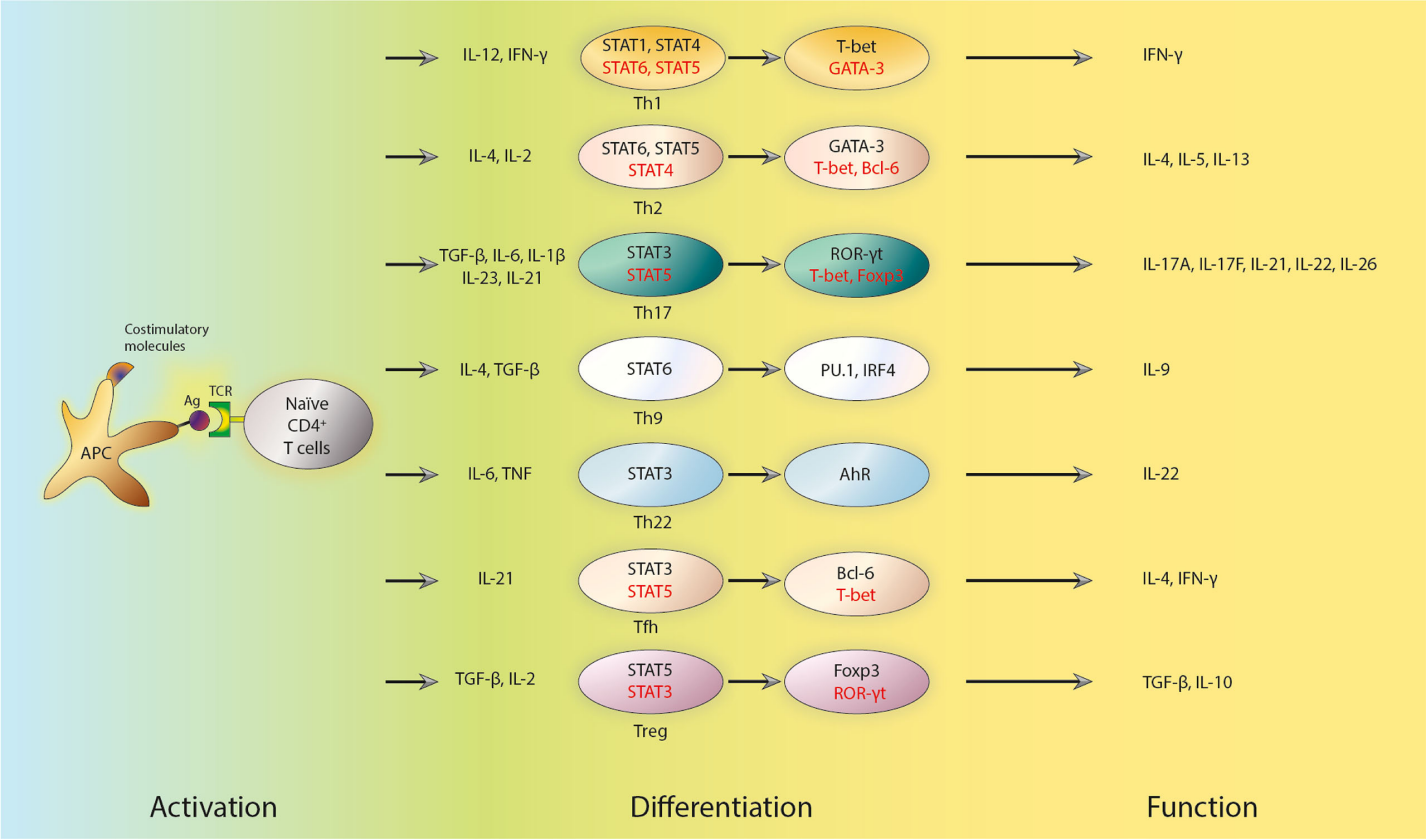
Subsets, transcriptional regulators and key cytokines of Th cells. APCs activate naïve CD4^+^ T cells by presenting antigens. The activation also requires combination with costimulatory molecules, which triggers the production of polarizing cytokines and subsequent differentiation of naïve CD4^+^ T cells into distinct effector Th and Treg subsets. The polarized differentiations are characterized by lineage-specific regulators and the secretion of key cytokines. The early response of those T cells is regulated by STATs. STATs can further influence the expression of master transcription factors. Positive regulators are shown in black, while negative regulators are shown in red. Th cells, T helper cells; APCs, antigen presenting cells; Treg cells, regulatory T cells; STAT, signaling transducer and activation of transcription molecules; Ag, antigen; TCR, T-cell receptor; IL, interleukin; TGF-β, transforming growth factor β; TNF, tumor necrosis factor; Tfh cells, follicular T helper cells; T-bet, T-box transcription factor 21; ROR-γt, retinoic acid-related orphan receptor γt; Foxp3, forkhead box P3; IRF4, Interferon Regulatory Factor 4; IFN-γ, interferon γ.

Antigen-presenting cells (APCs) activate naïve CD4^+^ T cells by presenting pathogen-derived peptides linked with MHC II, which, when combined with costimulatory molecules, increase T cell proliferation and the production of polarizing cytokines (7, 38), thus inducing naïve CD4^+^ T cells to differentiate into distinct Th subsets (**Figure 1**). T-cell receptor (TCR) signaling is essential for Th cell differentiation, a process wherein polarizing cytokines can induce the activation and/or up-regulation of certain transcription factors.

The role of TCR signaling in Th differentiation has long been investigated (39, 40). Soon after the report of Th1 and Th2 cells, it was discovered that peptide affinity as well as dosage, which could describe the strength of TCR signal, were pivotal factors in the differentiation of naïve CD4^+^ T cells (39, 40). In particular, low-dose peptide stimulation enhances Th2-cell differentiation (41). TCR signal strength also has a role in Th17 versus Treg cell differentiation (42). Treg cells prefer low TCR signal strength to initiate their differentiation, while Th17 cells need high TCR signal strength to differentiate (43–47). Pathogens also determine Th differentiation by altering TCR signal strength (48). The following studies have shed light on the mechanisms of how TCR signal strength affects Th cell differentiation. By activating specific signaling pathways downstream of TCR (42), TCR signal can mediate the expression of unique genomic programs including defined transcriptional factors and special epigenetic regulations (49). For example, interferon regulatory factor 4 (IRF4) regulates the differentiation of several types of Th cells, including Th2, Th17, and Tfh cells (50). cMyc and forkhead box transcription factor O1 (FoxO1) are also transcription factors (TFs) in the downstream of TCR signaling that play essential roles in Th cell differentiation (51, 52).

It is widely known that cytokines, in addition to TCR signaling strength, are essential for Th cell differentiation as well. Certain cytokines trigger the expression of lineage-specific master transcription factors. Indeed, and at least *in vitro*, IL-12 and IFN-γ can promote Th1-cell differentiation (53). APCs, such as macrophages and dendritic cells, release IL-12 which subsequently stimulates Th1 cell differentiation by activating the transcription factor signaling transducer and activation of transcription 4 (STAT4) (54, 55). Th1 cell differentiation is assisted by IFN-γ, which is generated by Th1 cells *per se* and activates STAT1 (56, 57). These regulatory events ultimately induce the cytoplasmic expression of the master transcription factor, T-box transcription factor 21 (T-bet), to regulate a variety of Th1-specific genes directly (53). According to a recent study, the transcription factor p73 could inhibit Th1 differentiation by negatively regulating IFN-γ production (58). STAT6 is activated by IL-4 and can further cause Th2-cell differentiation (59, 60). In addition to IL-4, IL-2 is also required for Th2 cell differentiation *in vitro* by activating STAT5 (41, 61). GATA binding protein 3 (GATA3) is a master transcription factor that regulate the differentiation of Th2 cells (62, 63), and it may promote Th2 cell differentiation through several mechanisms (64), including direct binding to the promoter region and regulating epigenetic modification of Th2-specific genes (65, 66). TGF-β, together with IL-6, IL-1β, IL-23, or IL-21, plays an essential role in promoting the expression of the master transcription factor retinoic acid-related orphan receptor γt (RORγt) and Th17 cell differentiation through activating STAT3 (67–69). However, STAT5 activation mediated by IL-2 inhibits Th17 cell differentiation (70). In multiple sclerosis, Qian et al. discovered that zinc-finger E homeobox-binding 1 (ZEB1), a transcription factor, enhances JAK-STAT4/3 signaling during Th1/Th17 differentiation by suppressing the production of a JAK2-targeting miRNA (71). TGF-β, retinoic acid, and IL-2 in the periphery promote Treg cell differentiation, and its master transcription factor is Foxp3 (72–74). STAT3 activation, probably by IL-21, is required for Tfh cell differentiation, but STAT5, which is activated by IL-2 decreases Tfh cell differentiation (33, 75). The master transcription factor for Tfh cell differentiation is Bcl-6 (76).

Th cell differentiation is a positive feedback loop reinforced by various cytokines. For instance, IFN-γ generated by Th1 cells could induce IFN-γ non-producers to secrete IFN-γ during Th1 cell differentiation. IL-4 secreted during Th2-cell differentiation can also cause IL-4 non-producers to express IL-4. Thusly, the differentiation of Th1 and Th2 cells are enforced by such positive feedback loops. TGF-1 and TGF-3 are both produced by Th17 cells and could act as positive signals for Th17 cell development (77). Apart from theses closed loop regulations, active transcriptional factors in one lineage frequently influence the expression of transcription factors implicated in other lineage decisions, which is called cross-regulation. T-bet overexpression, for example, reduces GATA3 function and decreases *Gata3* transcription (78). T-bet and RORγt (79) as well as Foxp3 and RORγt, have also been discovered to possess such cross-regulation properties (80, 81). Besides cytokines, metabolites can also profoundly influence the fate and functions of Th cells, which has been well reviewed elsewhere (82).

### Blood-Brain Barrier Permeabilization of Th Cells

Th cells perform diversified tasks in the CNS, and one prerequisite is their permeabilization through the blood-brain barrier (BBB). The BBB protects peripheral toxic substances and immune system elements from invasion and thusly maintains immunological homeostasis in the CNS. The BBB is primarily composed of brain endothelial cells that are tightly linked together by unique protein complexes, and it simultaneously exhibit a high enzymatic activity, which allows them to selectively transfer substances from the bloodstream to the CNS (83). Intercellular and vascular adhesion molecules such as intercellular adhesion molecule-1 (ICAM-1) and vascular cell adhesion molecule 1 (VCAM-1), as well as P- and E-selectin, are vital structures expressed by BBB endothelial cells. Those adhesion molecules, together with various cytokines, tightly regulate this guardian of the CNS.

However, peripheral immune system components are capable of crossing the BBB in several pathological conditions. ICAM-1 and ICAM-2 are involved in the migration of Th1 and Th17 cells across the BBB. ICAM-1/-2-deficient mice exhibited ameliorated symptoms of both conventional and atypical experimental autoimmune encephalomyelitis (EAE) caused by encephalitogenic Th1 and Th17 cells (84). Interestingly, it was proved that the tight connections of the BBB were disrupted when IL-17 and IL-22 attached to the corresponding receptors expressed on BBB endothelial cells (85). After recognizing antigens presented by APCs, activated Th17 cells were shown to reach the perivascular region and produce IL-17, which suggest that perivascular Th17 cells might affect BBB integrity and increase leucocyte migration (86, 87). Another study discovered that pro-inflammatory cytokines increased the synthesis of CCL2 and CXCL1 in brain endothelial cells, while CCL2, CCL5, CCL20, and IL-17 could stimulate Th17 cell migration (88). Th1 cells migrate slower than Th2 cells, owing to differed chemokine/chemokine receptor interactions (89).

## Th Cells in Neuroinflammation

### Autoimmune CNS Inflammation

**Figure 2:**
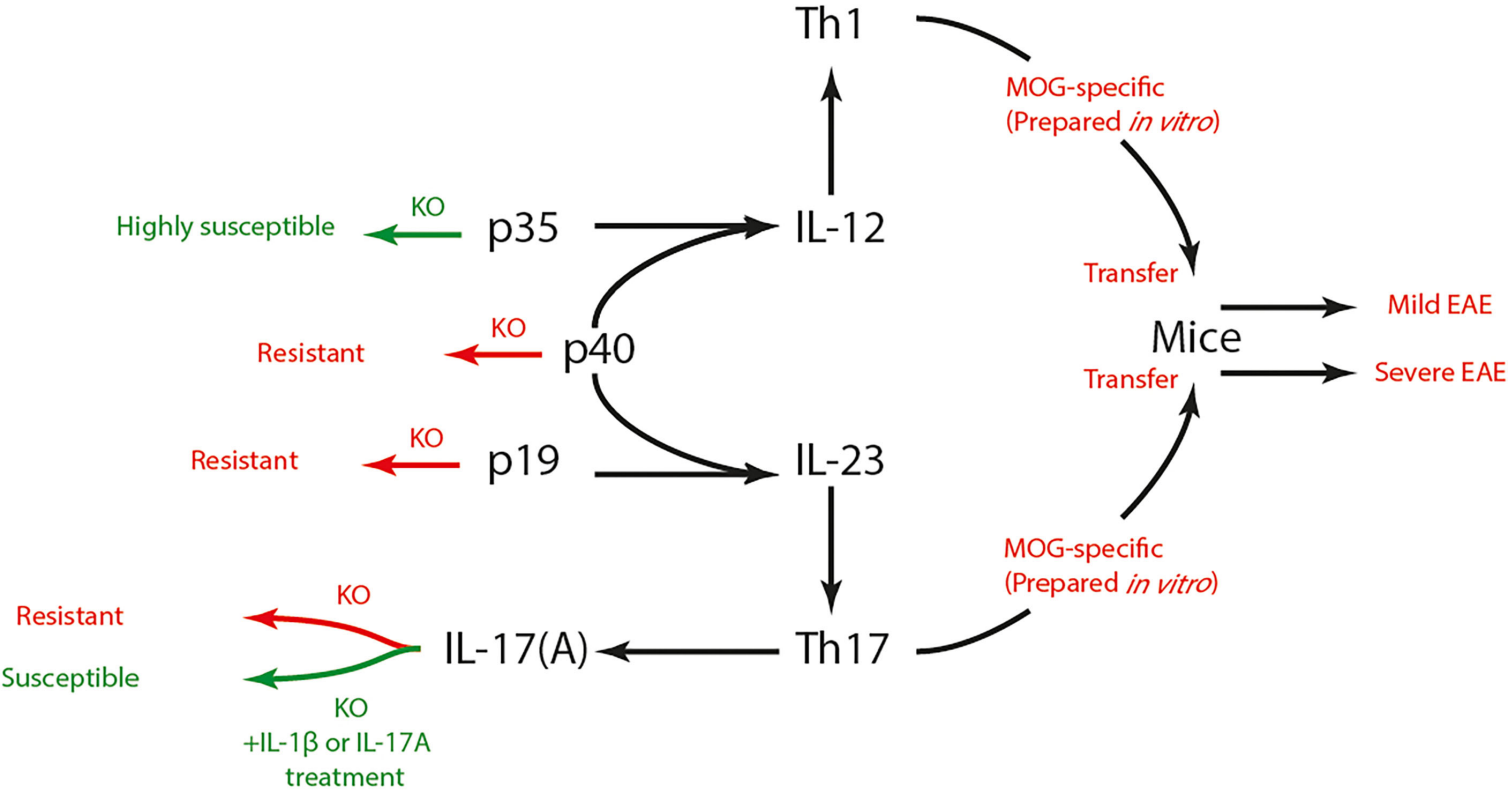
Th1, Th17 cells and EAE susceptibility. IL-12 and IL-23 respectively promotes the differentiation of Th1 and Th17 cells. IL-12 is composed of p35 and p40 subunit, while IL-23 is composed of p19 and p40 subunit. Knock-out of p40 or p19 induced resistance to EAE in mice, yet p35^-/-^ mice showed high susceptibility to EAE. IL-17 is the signature cytokine secreted by Th17 cells. IL-17-dificient mice also showed resistance to EAE, which could be reversed by IL-1β or IL-17 treatment. Both MOG-specific Th1 and Th17 cells could induce EAE after being transferred to mice, yet their severities differ. Th, T helper; EAE, experimental autoimmune encephalomyelitis; IL, interleukin; MOG, myelin oligodendrocyte glycoprotein; KO, knock-out.

The pathogenesis of autoimmune diseases results from a compromised immune tolerance toward a specific self-antigen. Multiple sclerosis (MS) is an autoimmune CNS disorder characterized by inflammatory demyelination and axonal transection (90). The worldwide prevalence of MS ranges from 5 to 300 per 100 000 people and increases at higher latitudes (91). The role of Th1 cells in MS pathogenesis has long been pronounced based on various studies with EAE animal models and MS patients. As a pro-inflammatory subset of Th cells, Th1 cells were found to abode and abound in brain lesions of EAE animals (92), as well as to cause M1-phenotype-oriented differentiation in CNS resident microglia (93). IFN-γ, which could be produced by Th1 cells, was also found abundant in brain lesions of MS patients (94). Subsequent evidence supplementarily questioned this Th1-dominance theory as the susceptibility to EAE still existed in animals with incomplete Th1 function (impaired IL-12 or IFN signaling) (95–97). After the discovery of Th17 cells (14) and with the understanding of IL subunits (98), it was proved that knockout of either the subunit shared by IL-12 and IL-23 (p40) (99) or the subunit exclusive to IL-23 (p19) (100), but not the subunit exclusive to IL-12 (p35) (95, 99), could induce resistance to EAE in rodents (**Figure 2**). Thusly, the conversion toward a Th17-dominance was initiated and has been well established nowadays. A study proved that knockout of IL-17A could induce resistance to EAE in mice (101). Actually, evidence also suggested that Th1 and Th17 might mediate distinct types of EAE, as transfer of either MOG-specific Th1 or Th17 cells prepared *in vitro* could induce EAE in mice although their severities differ (102). Taken together, the synergy of Th1 and Th17 may be the optimal description of the Th cell-mediated pathogenesis of MS/EAE.

**Figure 3:**
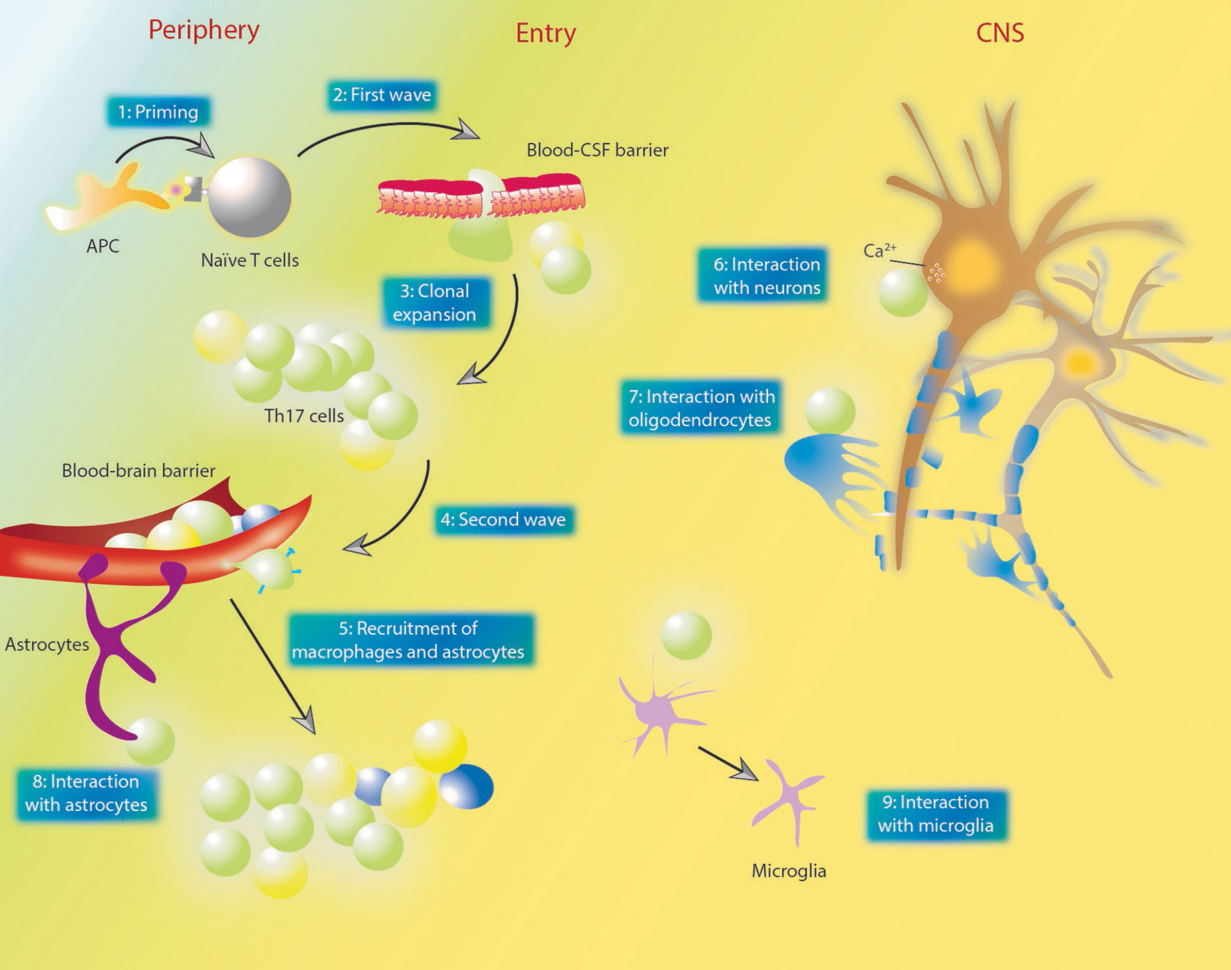
The two-wave theory of MS pathogenesis and the interactions between Th17 and other cells. **(1)** in the priming process, naïve T cells in the periphery become antigen-specific memory T cells. **(2)** those T cells infiltrate the blood-CSF barrier located in the choroid plexus. **(3)** after entering the subarachnoid space, Th17 cells undergo clonal expansion. **(4, 5)** the second wave denotes the activation of the BBB and recruitment of perivascular leukocytes. Plasma membrane molecules such as CCR6, CD6 and CD49d enables Th17 cells to cross BBB endothelium. Dendritic cells and macrophages could be recruited by GM-CSF secreted by Th17 cells. **(6)** Th17 cells contact neuron directly and lead to neuron damage *via* affecting intra-neuron Ca^2+^ concentrations. **(7)** Th17 cells could damage oligodendrocytes through direct contact or increased oxidative stress. **(8, 9)** various studies also demonstrated the effect of Th17 cells and their cytokines on astrocytes and neurons. GM-CSF could directly act on astrocytes and promote their recruitment. MS, multiple sclerosis; Th, T helper; CSF, cerebrospinal fluid; BBB, blood brain barrier; GM-CSF, granulocyte-macrophage colony-stimulating factor; APC, antigen-presenting cells; CNS, central nervous system.

Prior understandings of Th17 functions in autoimmune diseases such as MS/EAE, inflammatory bowel disease and psoriasis have been thoroughly reviewed (103–110). Vis-à-vis MS/EAE, a two-wave theory was proposed (111, 112) (**Figure 3**) that upon a process named priming, naïve T cells in the periphery become antigen-specific memory T cells (Th17 cells), and at this point, the first wave strikes during which those Th17 cells infiltrate the subarachnoid space through the choroid plexus (113). After being presented with antigens by APCs, those infiltrated Th17 cells undergo clonal expansion, and further induce a secondary wave characterized by the activation of the BBB, as well as the subsequent recruitment and infiltration of perivascular leukocytes.

Throughout the two waves, Th17 cells interact with other resident or recruited cells in the CNS to cause direct or indirect damages (**Figure 3**). T cells can enter the CNS through either the epithelial blood cerebrospinal fluid barrier (BCSFB) within the choroid plexus or the endothelial BBB (114). Actually, Th17 cells exhibit BCSFB permeability under both inflamed and non-inflamed conditions (114). Th17 cells also demonstrate superior BBB permeability compared to Th1 cells as they express certain surface molecules on their plasma membrane, such as CCR6, CD6 and CD49d, which mediate their interactions with endothelial cells (115). However, Broux et al. recently confirmed that IL-26, preferentially produced by Th17 cells, could enhance BBB integrity both *in vitro* and *in vivo*, and also reduce the severity of EAE (116). It was proved that Th17 cells could contact neurons directly, leading to neuron damage *via* affecting the intracellular Ca^2+^ concentration (117). On the other hand, neuronal activation was found to promote CCR2^+^CD4^+^ lymphocyte infiltration (118). Oligodendrocytes (OLDs), the most endangered cell type in MS/EAE, were also shown to be viciously influenced by Th17 cells, as evidenced by the fact that Th17 cells could promote oxidative stress-mediated OLD apoptosis (119), and in a more recent study, form prolonged stable contact with OLDs and thereby induce the release of glutamate and consequent demyelination in a CD29-dependent manner (120). IL-17-related signaling was also associated with the proliferation, differentiation and functioning of OLDs (121–123). Dendritic cells and macrophages could be recruited by granulocyte-macrophage colony-stimulating factor (GM-CSF) secreted by Th17 cells (124). Expressing IL-17 receptors, astrocytes are thought to be closely related to neuropathology in EAE (125), and astrocyte-specific silencing of IL-17 signaling could lead to EAE amelioration (126). A recent study confirmed that CNS-infiltrated CD4^+^ T cells could evoke a rapid and vigorous Ca^2+^ increase in astrocytes *via* promoting ATP release from glia (127). Withal, Th17 cells could also promote the maturation of B cells (128) and the formation of ectopic lymphoid follicles in target organs (e.g., brains in MS/EAE) (129), allowing for the production of antibodies (128). Apart from cellular types of heterogeneity, Th17 cells could also impact CD4^+^ T cell populations, as reported rather recently that IL-17 can directly act on non-Th17 effector CD4^+^ T cells to induce resistance to immunosuppression by CD8^+^ T cells (130).

However, the regulations and functions of Th17 cells are not constant during MS/EAE. It was reported that IFN-β, a clinical treatment for MS patients, had inflammatory effects in Th17-induced disease through the production of IL-6 (131). Some studies also suggested that IFN-β inhibited the differentiation and function of Th17 cells (132). Agasing et al. reported in a recent article that the impact of INF-β on Th17 cells is temporal (133). During early Th17 development, IFN-β inhibits IL-17 production, yet during late Th17 differentiation, IFN-β synergizes with IL-23 to promote a pathogenic T cell population with both Th1 and Th17 characteristics that expresses elevated levels of the potent inflammatory cytokines IL-6 and GM-CSF, as well as their transcription factor BLIMP. Certain types of phenotypic plasticity of Th17 cells are endowed by the intricate regulation network of the adaptive immune system. Th17 cells are able to acquire phenotypes similar to Th1 cells (secreting IFN-γ) (134, 135), Treg cells (secreting IL-10) (136) or Tfh cells (secreting CXR5, ICOS and Bcl-6) (129). However, plasticity is not a trait exclusive to Th17 cells amongst all Th subsets. Regulatory T helper (Tfr) cells could exhibit Th1-, Th2- or Th17- like phenotypes (137), and natural Treg cells could convert to Th17 cells (138) under certain circumstances.

In normal physiological conditions, Th17 cells work synergistically with other Th cells to maintain immunological homeostasis. Accumulating evidence has shown that balances between subsets of Th cells are sabotaged during autoimmune disorders, including MS/EAE. The dichotomy of Th1 and Th2 cells was orchestrated by another dichotomy of Th17 and Treg cells after the discovery of Th17 in 2005 (14). The pro-inflammatory roles of Th1 and Th17 cells, as well as the anti-inflammatory roles of Th2 and Treg cells in the pathogenesis of MS/EAE were well examined (139, 140). A phase 1 study on the administration of Treg cells for 14 relapsing-remitting MS patients was reported recently (141). Another imbalance between Tfh and Tfr cells were also reported (142). IL-9, a cytokine mainly secreted by macrophages, microglia and CD4^+^ T cells in the brain, was confirmed to have beneficial effects in MS (143, 144), as they decrease the activation state and promote the anti-inflammatory functions of macrophages (144). Later, it was also reported that IL-9 could mitigate EAE *via* suppressing GM-CSF production by CD4^+^ T cells (145). Of note, a new subset of Th cell, named ThGM, was reported recently (37). This subset was characterized by the expression of GM-CSF but lacked expression of signature transcription factors and cytokines of established Th lineages. The study also proved that EAE mice had increased numbers of ThGM cells in both the periphery and CNS, and the encephalogenicity of ThGM cells requires T-bet signaling.

In sum, the proinflammatory contribution of Th17 and Th1 cells, as well as the anti-inflammatory contribution of Th2 and Treg cells have been well recognized in MS/EAE. However, as more Th cell subsets emerge, the interaction or transformation between these subsets, and the complex regulatory network of adaptive immunity all point to a broader perspective of understanding the primary drivers of MS/EAE. With more precise control over these regulatory pathways, superior therapeutic interventions could be further achieved.

### Neurodegeneration

As a result of increasing numbers of senior people, the global burden of senescence-related neurodegenerative diseases has been underscored (146, 147). As the most common cause of dementia, Alzheimer’s disease (AD) was estimated to affect around 100 million of the population worldwide by the time of 2050 (148). The major pathological change of AD is the formation of extracellular Aβ plaques and intracellular neurofibrillary tangles (149). These abnormal neurofibrillary structures may be related to the abnormal phosphorylation state of tau proteins. The exact mechanism of AD pathogenesis is still elusive, yet neuroinflammation involvement has been implicated, in that altered cytokine milieus, CNS and peripheral lymphocyte profiles, as well as their activation state were reported in both AD patients and animal models. Moreover, Th cells also play controversial roles during the attempt of vaccine development against AD.

Altered cytokine levels in AD patients and animal models have been widely reported. It was suggested and validated that, although the prominent pathological changes occur in the CNS, AD is still a systemic inflammatory condition which incurs changes of cytokine levels in the periphery. Compared to normal groups, AD animals showed in the brain, nasal tissue, spleen and cervical lymph nodes elevated levels of IFN-γ and IL-17, but not IL-4 (150). The serum of AD patients was shown to contain higher levels of IL-17 and IL-23 (151), suggesting a possible Th17 polarization. Also, the amount of circulating immune cells that produce IL-17, IL-6 and IFN-γ was increased in AD patients (152). Moreover, the increase of peripheral Th17 cell proportion was correlated with the degree of amyloidopathy (153) in AD patients, as well as early AD (154). Treg cells, or cytokines related to Treg cells, on the other hand, were shown to be down-regulated in AD (155, 156), and the Th17/Treg ratio was also related to the disease state (157), with Treg proportions positively related to neurodegeneration markers (153). Although Th cell subpopulation may alter during AD, a study suggested that the total number of CD4^+^ and CD8^+^ T cells showed no significant change in general (152). The crosstalk between CNS changes in AD and peripheral inflammatory responses could also be validated by the fact that AD could exaggerate the natural shift of serum cytokine profiles from Th1-related dominance toward others during senescence (158). Actually, the impact of AD on the peripheral immune cells seems more profound considering that naïve T cells isolated from AD patients secret more proteins which include IL-21, IL-6, IL-23 and RORγt (159). A recent study found tau-specific CD4^+^ T cell responses in both AD patients, age-matched controls, and even in young controls (160).

The aforementioned studies present vital understandings and hints on how Th cells and the cytokines that they secrete may participate in the systemic inflammation of AD. The crosstalk between peripheral immunocytes and AD neuropathology might also be mediated by gut microbiota composition change (161), which was observed in both AD patients and animal models, or more directly, *via* the invasion of Th cells into the CNS (162–164). It has been proved that a significant amount of peripheral lymphocyte could infiltrate the CNS in the pathological setting of AD (165) and Th17 cells, for instance, could achieve throughfare *via* CCR6-CCL20 signaling (166). After infiltration, Th17 cells further promote neuroinflammation and neurodegeneration by secreting cytokines, or else cause damage to neurons by direct contact and Fas-FasL signaling which induces autophagy (167–169). GM-CSF, mainly secreted by Th17 cells, could further enhance the function of microglia to act as APCs for presenting Aβ to T cells and priming T cell activation in AD (170).

Participation of Th cells in AD is not restricted to Th17 cells, as the pro-inflammatory role of Th1 cells, anti-inflammatory role of Th2 cells that could induce the production of anti-amyloid antibodies as well as the roles of Treg cells were also described in AD (171–176), with one study even finding that Th2 cells could benefit AD amelioration without CNS infiltration (177). Interestingly, one study reported destructive effects of Th2 responses in brain aging (178), and another study found that transient depletion of Treg cells in AD mice could induce clearance of Aβ plaques as well as reversal of cognitive decline (171).

Several studies focused on interfering with CNS Th cells or their correspondent cytokine levels as an attempt to investigate their participation during the pathogenesis. Although an elevated level of IL-17 is a natural response observed in AD patients and animal models (167, 179, 180), deliberate altering IL-17 levels incurred controversial results. Silencing IL-17 production in AD mice through AAV intracranial injection resulted in amelioration of AD symptoms and a significant increase of ABCA1 (181), which is a transporter of Aβ from brain to blood. Cristiano et al. reported that neutralization of IL-17 could rescue neuropathological changes and memory impairments in AD (182). However, Tfilin et al. reported that intravenous administration of IL-17 to mice could modulate neurogenesis and improve learning (183). Intracerebroventricular (ICV) transfer of Aβ-specific Th1 and Th17 clones in APP/PS mice worsened pathological condition through downregulating Treg cells in the periphery and CNS (184). In another study, ICV injection of Aβ-specific Th1 cells in 5XFAD mice promoted the differentiation of an MHCII^+^ microglia subset and was suggested to play a key role in AD pathology (185). Similarly, ICV transfer of mixed Aβ-T cells resulted in decreased pathology (186) yet transfer of Aβ-Th2 was evidenced to cause no obvious inflammatory effect (164) or could alleviated burden (177). However, Eremenko et al. recently reported that engineered BDNF-expressing Aβ-specific CD4 T cells, upon ICV injection to the 5XFAD mouse model of AD, could mitigate both amyloidopathy and CNS inflammation (187).

As AD remains a disease affecting millions of people with still no cure, the research for possible therapeutic targets and optimal vaccination choices is still thriving. The first clinical trial of vaccination was aborted because a small but significant portion of patients showed severe encephalitis due to strong Th1 responses to all Aβ (188). Since then, researchers suggested that qualified vaccines against AD shall avoid strong activation of Th1/Th17 cells while promote Th2 cells (189), which could be possibly achieved *via* formulating with adjuvants that induce effective Th2 responses (190). Donepezil, an acetylcholinesterase inhibitor that’s commonly used as treatment for AD, was proved to possess immune-modulating effects (191).

Parkinson’s disease (PD), which was proposed to be the fastest growing in prevalence, disability and deaths among certain neurological disorders (192), is another neurodegenerative disorder that is estimated to affect millions of people worldwide (146) and has also been related to autoimmune responses, especially in its early stages (193, 194). The signature neuropathology of PD is dopaminergic neuron death in the substantia nigra pars compacta, which is characterized by abnormal accumulation of Lwey bodies and α-synuclein, as well as the initiation of a series of neuroinflammatory responses (195–197).

Studies on Th cells and their cytokines in the field of PD mainly include those focusing on their roles in the death of dopaminergic neurons (198), as well as those reporting or explaining the dynamic changes of Th cells during PD (199, 200) and their correlation with clinical parameters (201). *In vitro* experiments proved that α-synuclein could promote dendritic cell-induced polarization of CD4^+^ cells toward Th1,Th2 and Th17 subsets (202), and the *in vivo* accumulation of α-synuclein could also upregulate Th17-related immune response through molecular chaperones and cytokines such as IL-6 and TNF-α (203), or through microglia activation and M1-oriented differentiation (204). Similar to the case in AD, reactive T cells are also able to infiltrate the CNS during PD (205–208), or even years before presentation of motor symptoms (209), which is the major diagnostic criteria of clinical PD. In fact, established evidence reveals that peripheral T cell reactivity to α-synuclein indeed starts early before clinical symptoms are present, yet those responses start to drop after they peak around diagnosis (209). The specific subset of those reactive T cells was not identified in the original report. An intractable string of evidence from PD patients demonstrates that the Th17 cell levels could be increased (210, 211), decreased (212) or remain unchanged (213, 214), and admittedly, methodological variation exists in those different studies. Nonetheless, various studies on patients or MPTP-induced animal models showed applicable facts of increased Th17 and Th1 cells which might be pro-inflammatory (215, 216), decreased Treg cells which might be anti-inflammatory (212, 215, 217), disrupted Th1/Th2, Th17/Treg, as well as Tfh/Tfr balance in PD (218, 219).

Established evidence also provided a preclude to explorations into the exact mechanisms of Th cells’ mediating neuroinflammation in PD. Liu et al. showed in MPTP-induced PD mice of different genotypes that dopamine receptor 2 (DR2) or CD4-specific DR2 knock-out, instead of DR1 knock-out, could exacerbate phenotypical, pathological and inflammatory changes related to PD (220). González and colleagues also demonstrated that mice bearing DR3-deficient CD4^+^ T cells were refractory to MPTP-induced neuroinflammation and neurodegeneration (221). Liu et al. evidenced that blocking either ICAM-1 in VM neurons or LFA in Th17 cells could abolish Th17-induced neuronal death (222). Th2 cells, on the other hand, were comparatively less reported. Kustrimovic et al. reported reduced Th2 cells in the peripheral blood from PD patients (212). In one study, the effect of IL-13 and IL-4 on oxidative stress to dopaminergic cell lines were investigated (223). They found that neither IL-13 nor IL-4 could alter cytotoxicity. It was also reported that mice lacking IL-13Rα1 was protected from neuronal loss when compared to littermates (224, 225), suggesting a neurotoxic role of its ligand IL-13 and or IL-4.

Of note, amyotrophic lateral sclerosis (ALS) is considered as a multisystem neurodegenerative disorder, and transgenic mice overexpressing mutant Cu-Zn superoxide dismutase 1 are utilized as the corresponding animal model. A study using these mice identified a protective role of Th cells, as mice lacking CD4^+^ T cells demonstrated exacerbated disease progression and increased mRNA levels of proinflammatory cytokines (226), thus implying a beneficial role of Th cells.

Neurodegenerative diseases, compared to other CNS disorders discussed herein, relate closely to senescence. Common topics concerning studies on neurodegenerative diseases include the accumulation of specific antigens as well as neuronal loss. Although the pro- or anti-inflammatory impressions on Th cells have been generally established, their roles in neurodegenerative diseases still seem controversial. Apart from the differed study results reported by different research groups, the functions of a specific Th subset may appear conflicting.

### Epilepsy

Epilepsy is a spectrum of brain disorders marked by an enduring proclivity for epileptic seizures, as well as the neurobiological, cognitive, psychological, and social repercussions of this condition (227). Epilepsy affects millions of people worldwide, and it most usually begins before the age of 1 and increases after the age of 50 (228). The established hypothesis of epileptogenesis is focused on the excitatory-inhibitory imbalance in the central nervous system. However, numerous studies have suggested the possible roles of neuroinflammation in epileptogenesis (229, 230).

Several original or meta- analysis on epilepsy patients reported up-regulated IL-1α, IL-17, IFN-α, CCL4, CCL11, CXCL10, CX3CL1, HMGB1, bFGF and down-regulated CD4^+^ percentage in the peripheral blood (231, 232). Up-regulated IL-1α, IL-17, IL-13, CCL2, CCL5, CCL19 and CCL22 solely in the brain (231), as well as up-regulated IL-1ra, IL-1β, IL-6 and CXCL8/IL-8 ratio in peripheral blood, CSF and brain were also suggested (231). However, it’s noteworthy to mention that, unlike other neurological disorders discussed herein, epilepsy is characterized by a continuum of ictal events (although there might be relapses in MS, the ictal events in epilepsies are rather temporary). The collateral pre-, post- as well as inter-ictal physiological fluctuations and the subsequent stress-induced responses might be comprehended as the consequences caused by epilepsy *per se* (233, 234), thus studies specifying the timing of sample harvesting were also conducted. For instance, it was found that the postictal levels of IL-6 and IL-1ra were significantly increased in the plasma of epilepsy patients (235–237), suggesting a possible affecting or causing role of these cytokines in ictal events.

Despite the enduring development of antiepileptic medications over the last few decades, more than 30% of patients with epilepsy have progressed to drug-resistant epilepsy (DRE), resulting in a considerable increase in epilepsy morbidity and death (238). The involvement of neuroinflammation in DRE has also been investigated to identify the possible relevance between immunological changes and drug resistance. Based on their findings from the peripheral blood of pediatric DRE patients that Th17 cell percentage and the expression of IL-17A and RORγt were significantly higher, while the proportion of circulating Tregs and expression of Foxp3, GITR, CTLA-4 were significantly lower, yet these alterations could be further reversed by ketogenic diet (which is a proved treatment to DRE), Ni et al. concluded that Th17/Treg imbalance is characteristic of childhood DRE, and this imbalance may contribute to DRE pathogenesis (239). Kumar et al. reported in the peripheral blood of pediatric DRE patients a change in CD4^+^ and CD8^+^ T cell subsets toward IL-17 production, further implying the participation of IL-17 in the pathogenesis of DRE (240). It has been confirmed that CD4^+^ T cells can infiltrate epileptic lesions located in the brain parenchyma (234). Xu et al. provided direct evidence that IL-17- and GM-CSF-producing γδT cells were concentrated in epileptogenic lesions from brains of DRE patients, and their numbers were positively related to disease severity, although the numbers of Treg cells were inversely related (241). Other studies involving pediatric DRE patients suggested significant changes of intracellular IFN-γ concentration among CD4^+^ T cell populations (242), differentiated immunological parameters caused by medication choices (243), and one study on DRE patients also confirmed higher levels of PD-1 in the serum and CSF (244).

The most prevalent kind of DRE referred for epilepsy surgery is temporal lobe epilepsy (TLE), which often responds well to brain surgery (245). Direct evidence connecting Th cells and epileptogenesis or epilepsy remains scarce. However, serological studies suggest that distinct changes in peripheral cytokine profiles could either be the cause or effect of epilepsy. As compared to controls, patients with TLE were reported to show significantly increased (235, 237, 246–249), marginally increased (231) or not increased (231) levels of IL-6, significantly increased IL-5 levels (246), as well as a decreased postictal IL-1ra/IL-1β ratio (231). One study using blood samples from 20 TLE patients together with 19 controls suggested a negative correlation between the frequency of CD4^+^ T cells and the age of seizure onset (250). Cytokines or chemokines, including IL-1α, IL-1β, CCL-1, CCL3, CCL-4 and CCL5, were also proved to be up-regulated in brain tissues from TLE patients (251–253). Of note, CCL3, CCL4 and CCL5 expressions were also identified in TLE neurons instead of normal control neurons (253). Studies on immunocyte populations or functions also revealed epilepsy-related difference. From the cytological aspect, higher expression levels of HLA-DR, CD69, CTLA-4, CD25, IL-23R, IFN-γ, TNF and IL-17 in CD4^+^ lymphocytes were identified in TLE patients (250).

TLE is further subdivided according to different clinical or pathological manifestations. One study concerning drug resistant-TLE suggested that those patients could be subdivided into two groups based on whether there was a peripheral increase of CD4^+^CD38^+^ cells (246). Another study on both TLE-limbic encephalitis (LE) and TLE-nonLE patients revealed that there was a higher ratio of CD4/8^+^ T cells in the peripheral blood of patients with TLE-LE as compared to TLE (254). Also, the study correlated a comparatively low ratio of CD4/8^+^ T cells with a blood-CSF barrier dysfunction in patients with TLE-LE. Hippocampal sclerosis (HS) is a neuropathological diagnosis, defined as severe hippocampal neuronal loss and gliosis (255, 256). HS is a well-known cause of TLE and is frequently linked to seizure resistance. Several studies using blood, CSF or brain samples from TLE-MS patients shed light on distinct neuroinflammatory profiles associated with HS. It was reported that there existed a postictal decrease of peripheral CD4^+^ cell count by 13% (257), while this decrease was more pronounced in patients with HS. The aforementioned increase of IL-1β in TLE patients was also more pronounced in HS patients (258, 259). Another study found increased frequency of CD4^+^ T lymphocytes expressing IL-6 in the peripheral blood of mesial TLE patients with HS when compared to healthy volunteers (260). As hippocampal sclerosis is an indication for epilepsy surgery, studies utilizing surgically resected HS tissues also suggested peripheral CD8^+^ and/or CD4^+^ T cell infiltration into the hippocampi (261), the perivascular region (262), or diffusely in the brain parenchyma (262). However, Lu et al. suggested rare CD4^+^ T cell infiltration into the hippocampi from 30 HS patients (263). One of these studies also showed a positive correlation between the number of infiltrated CD8^+^ and CD4^+^ cells into the sclerotic hippocampi (263).

The discovery of animal models mimicking human epilepsy enabled further understanding of epileptogenesis, as well as the development, screening, and evaluation for potential anti-epileptic drugs. Genetically epilepsy-prone rats (GEPR) were identified to possess a stable genetic predisposition to audiogenic seizures. De Sarro et al. showed a predominance of Th cells over CD8^+^ cells both in spleen and lymph nodes from GEPR-9s that were previously subjected to acoustic stimulation (264), suggesting that the altered T cell function could be attributed to neuroendocrine modulation. Electrical stimulation could also be adopted to induce seizures in rodents. Silverberg et al. utilized extracranially placed electrodes to potentiate seizures in mice, and they observed peripheral lymphocytes (including Th cells) infiltration into the brain 24 h after a maximal seizure, which peaked at 48 h and became undetectable at 7 d (234). Avdic et al. utilized intracranial electrode to induce non-convulsive status epileptics, a prolonged epileptic seizure with subtle symptoms, in rats (265). They reported increased levels of IL-6 in both brain and serum 6 h after non-convulsive epileptic seizures, and after 4 weeks when 75% of those rats exhibited spontaneous seizures (SS), they further compared those rats with SS to both those without and unstimulated rats, finding a decrease of CD4^+^ T cells in the peripheral blood. Some studies using the kainic acid model of status epileptics suggested a possible role of Th cells in epileptogenesis. Xu et al. reported ameliorated seizure activity in both γδ T cell and IL-17RA deficient mice, as well as in recipients of Treg cells, while Treg depletion exacerbated seizure severity (241). However, Deprez et al. showed that depletion of CD4^+^ and/or CD8^+^ T lymphocytes by targeted gene deletion results in a marked shortening of the delay prior to seizure onset, and also demonstrated the worsening of epileptic neuropathology due to CD4^+^ T cells transferred in MHCII-knockout and RAG1-knockout mice (266). *In vitro* experiments showed that both IL-17 and GM-CSF induced neuronal hyperexcitability in brain slice cultures (241).

Based on the preceding discussion and existing evidence, conclusive remarks on the roles of Th cells in epilepsy or epileptogenesis are yet challenging to address due to the following reasons. The term “epilepsy” intrinsically denotes a spectrum of disorders with heterogeneous etiology (267), and genetic as well as environmental factors are considered relevant to the pathogenesis. Therefore, epilepsy disorders with different etiologies or clinical/histopathological manifestations may inherently involve immunological alterations to differed degrees. This may also explain the above-listed incongruent results. Also, in most epilepsy disorders, no specific antigens can be identified. As a second thought, the “cause or effect” role of Th cells in epileptogenesis may require a re-evaluation. Nonetheless, the participation of Th cells during epilepsy has become an evident truth.

### Traumatic Brain Injuries

Traumatic brain injuries, characterized by mechanical damage to the parenchyma and/or meninges, are ensued by a series of neuroinflammatory responses including the infiltration and activation of a distinct spectrum of immune cells, as well as the secretion of certain cytokines and growth factors. The participation of these immune cells and molecules are not only destined to restore homeostasis, but also exacerbate inflammation in some cases.

The direct and primary damage in TBI is the mechanical force or contusion exerted on the brain parenchyma. Upon exertion, small blood vessels experience a shear injury, leading to BBB dysregulation (268). The secondary injuries of TBI usually denote those consequential injuries due to inflammatory and metabolic responses. Cellular infiltration to the lesion usually pursues blood brain barrier breakdown, with the infiltrate comprising T cells, neutrophils and macrophages (269, 270). Forsooth, evidence is that leukocytes begin to adhere to the CNS endothelium hours after the injury (271).

One aspect of evidence concerning Th cells in TBI that attracts researchers’ attention is that an intricate and highly regulated array of interleukins exists in the whole pathological duration after the initial injury. Cytokines, including those mainly associated with Th1/2 cells (IL-2, IL-4, IL-6, IL-10, IL-12, IFN-γ) and with Th17 cells (IL-17), are differentially regulated in the acute and chronic phases of either clinical or experimental TBI, as excellently reviewed by Bao et al. in detail (272). Moreover, some of these immunological parameters were also clinically associated with the severity of the injury (273), the recovery of certain neurological impairments (274), or even the overall prognosis (275, 276). Downregulation of IL-2/sIL-2R ratio was observed in clinical TBI (277), and a specific downregulation of both IL-2 and sIL-2R was also identified during 10-50 days post trauma (278). IL-6, the level of which peaks at 6 h after injury (279), were also indicated as a prognostic criterion (280). However, different research results revealed seemingly controversial roles of IL-6 in the pathological changes after TBI. Some suggested that high levels of IL-6 incurred and exacerbated brain damage (280), whereas some also suggested a neuroprotective function of IL-6 in the healing process (281). This evidence leads to a hypothesis that IL-6 may be related to increased inflammatory response after TBI, since IL-6 deficiency was shown to cause poor behavioral performances in animal models (282). The peak of IL-10 occurs later when compared to IL-6 (at 24 h) (279), and it maintains an elevated level throughout the acute phase of TBI (283). IL-12 was shown to be upregulated for a post-trauma time course of 14 days (284). Th17 cells were also correlated with the secondary pathogenesis of TBI. The major cytokine secreted by Th17 cells, namely IL-17, was described to be upregulated after TBI (285). Considered as a distinct type of Th cells, Treg cells are also indicated in the secondary damage after TBI, especially when considered together with Th17 cells (286). Treg cell levels are described to relate to the degree of neuro-recovery in both human and animal models (286, 287). Transfer of Treg cells into TBI animal models also demonstrates a neuroprotective result (288). The Th17/Treg balance has a significant impact on the pathogenesis of neuroinflammatory diseases such as MS and EAE in animals, and the increase in the ratio of Th17 cells to Treg has also been related to higher injury severity of TBI (286).

Another prominent change after TBI is the activation of microglia, manifested as the preferentially polarization toward the M1 phenotype (289–292). Unlike type-2 microglia which secret regenerative cytokines and growth factors such as TGF-α, IL-10, BDNF, GDNF (293), type-1 microglia are capable of the release of proinflammatory cytokines including IL-1β, TNF-α, IL-6, etc. and the consequent obstruction of neurorepair events (289). As mentioned, IL-1β and IL-6 are crucial to the differentiation toward Th17 cells. Apart from the differed humoral milieu, M1-oriented, instead of M2-oriented polarization, could also incur Th1-like responses in T cells, during which the secretion of cytokines such as IFN-γ could in turn offer positive feedback to the M1 activation (93). Through the activation of SATA1 signaling, inflammatory M1-type microglia secrete CXCL10, which is the ligand for CXCR3^+^ Th1 cells, thus resulting in further white matter injury (292).

In summary, Th cells participate in the regulation of immune responses during the secondary damage of TBI. Infiltrated and activated Th cells may fulfil their responsibilities through preventing infectious complications, but may as well cause severe inflammatory damages that exacerbate disease progression and delay neuroregeneration. Based on more profound understandings of the complex immune regulation network, especially during the secondary damage, precise instructions on clinical interventions and therapeutics methods may be obtained.

### Mental Disorders

Neuroinflammation plays an important role in mental disorders. In recent years, the role of Th cells, especially Th17 cells, in mental disorders has attracted much attention. Different subsets and cellular products of Th cells have different impacts on the CNS. In general, Th17 cells are likely to play a deleterious role, while Treg cells usually have a protective effect. The imbalance between Th1 and Th2 cells is also a pathogenic factor in many mental disorders.

Depressive disorder is a general term denoting a group of diseases characterized by depressed mood. Monoaminergic system is the main target of drug therapy for depressive disorder, but about 30% of patients have poor response (294). A large number of studies reported the changes of immunocytes and cytokines in depression (295). Given that most of the altered cytokines are associated with Th cell differentiation (296), researchers have begun to focus on the role of Th cells in the pathogenesis of depression.

Changes of Th cells and their specific subsets were described in the setting of depression. Based on the results of one study, there were more CD4^+^ T cells and a higher level of IL-6 in the peripheral blood of depression patients (297). Nevertheless, no increase in CD4^+^ T cells was observed in postpartum depression patients compared to healthy postpartum women (298). Later-life depression females also showed a downregulation of CD4^+^ T cell-related genes (299). The major effector Th cell subsets involved in the pathogenesis of depression were reported to be Th1, Th17, and Treg cells (300). There seems to be a Th1 skewing in the Th1/Th2 ratio in depression (301), despite inconsistent findings regarding changes in IL-4 and IFN-γ levels in patients with depression (302–307). The frequencies of CD4^+^CCR7^low^T_CM_ cells, mainly Th1-like cells, were identified to be robustly correlated with MS-associated depression, deepening the understanding of inflammatory characteristics of depression (308). As for Treg cells, they are likely to exhibit a protective role in depression (295, 309). However, there are a few seemingly contradictory findings. Obermanns et al. found that the number of CD4^+^CD25^+^ Treg cells in the blood of depression patients decreased after psychological and pharmacological therapy (310). Additionally, patients with obesity and comorbid depression displayed a higher Treg cell proportion compared to non-depressed patients with obesity (311).

Various studies focused on the role of Th17 cells in depression (295). In a study that involved 40 patients with major depressive disorder and 30 healthy controls (312), an increase in peripheral Th17 cell number and a decrease in Treg cell number were found in patients. In addition, those patients demonstrated a higher level of RORγt mRNA expression and serum concentration of IL-17. Animal models also provide potent evidence on the participation of Th17 cells in depressive disorder. Beurel et al. analyzed the behavioral performance of mice treated with Th17 cells, CD4^+^ cells, or vehicle and RORγt (+/GFP) mice or mice administered RORγt inhibitor SR1001 or anti-IL-17A antibodies (313). They found that the number of brain-infiltrated Th17 cells was increased in mice after learned helplessness and chronic restraint stress training. Th17 cell intervention potentiated learned helplessness, while mice with Th17 cell dysfunction, induced by RORγt inhibition or anti-IL-17A antibodies, were relatively resistant to it. These studies indicate a pathogenic role of Th17 cells in depression. Further evidence is gained in recent studies on the role of Th17 cells in patients with a comorbidity of depression and an allergic or autoimmune disease (303, 314). Although these studies provide good models to study the relationship between depression and immunity, they have limitations of insufficient sample size and being a retrospective study in nature.

On the contrary, downregulation of Th17 pathway was reported in other studies (315, 316). Inconsistent results also appear in clinical studies and drug intervention studies (295). Therefore, the role of Th17 cells has not been totally confirmed. In spite of an excellent integrative model proposed by Anastasiya Slyepchenko et al. (301), knowledge gaps still exist in how Th17 cells induce neuroinflammation, the origin of Th17 cells in the CNS and the influence of gut microbiota on Th17 pathway. In this sense, large sample prospective studies and high-quality preclinical studies are needed.

Schizophrenia is another severe mental disorder. The etiology and pathogenesis of the disease are unknown, which may be related to heredity, neurodevelopment, neurobiochemistry and psychosocial factors, etc. Th1 and Th2 responses in schizophrenia have been excellently reviewed by Markus J. Schwarz et al. (317). In general, those studies point to a hypothesis: schizophrenia patients with predominantly negative symptoms and/or treatment resistance show a Th2 shift. Akkouh et al. reported a downregulation of mRNA expression of FOXP3 (a Treg-specific marker) and indicated an abnormal astroglia-CCL20-CCR6-Treg axis in schizophrenia (318). Another study regarding Treg cells provided supporting evidence, showing a reduced level of Treg cells when stimulated and elevated levels of proinflammatory cytokines (319). Mutant Disc1-L100P mice, a genetic model of schizophrenia, displayed an increase in CD3^+^CD4^+^ Th cells and a decrease in CD3^+^CD4^+^CD25^+^ Treg cells (320). By contrast, mutant Disc1-Q31L mice showed an increase in CD3^+^CD4^+^CD25^+^ Treg cells (321). In schizophrenia patients, the increase of CD4^+^ T cells was reported as well (322).

In recent years, studies on the association of schizophrenia with gastrointestinal inflammation, encephalitis and human endogenous retroviruses, along with studies on the role of Th17 cells in other mental diseases have brought to the fore the potential role of Th17 pathway in schizophrenia (323), about which contradictory results have been reported. An increased Th17 percentage was observed in schizophrenia patients and there was a positive relationship between the proportion of Th17 cells and psychotic symptoms (324). Similar results were reported in 22q11.2 deletion syndrome patients with psychotic symptoms (325). Studies on transcription factor signaling pathways confirmed the upregulation of Th17 pathway (326, 327). However, a decreased level of IL-17 and ratio of IL-17/TGF-β and an increased level of IL-4 and IL-27 (Th17 suppressing cytokines) in patients with schizophrenia were demonstrated in another study (328). Dimitrov et al. also found that the level of IL-17 was decreased in schizophrenia patients (329). It is likely that the distinctions between these findings are related to sample size, course of disease, types of cytokines, age, sex and other factors (323). Debnath et al. elucidated the interaction between Th17 pathway and dopamine system as well as prenatal infection and maternal immune activation (MIA) in schizophrenia (323). Moreover, they pointed out that Th17 cells played a “sinner” role in schizophrenia by destroying the blood-brain barrier, invading the CNS, and causing neuroprogression through neuroinflammation along with other cytokines and microglia. In-depth clinical and preclinical studies are needed to uncover the exact role of Th17 pathway in the pathogenesis of schizophrenia.

Autism spectrum disorders (ASDs) are a group of neurodevelopmental diseases characterized by impaired social interactions, communication deficits and stereotypic repetitive behaviors. The development of autism is closely related to immune aberrations. For instance, DiStasio et al. reported a correlation between cytotoxic astrocyte blebs and T cells in the postmortem brains of ASD patients (330). Dysregulation of HLA-DR, Helios, IL-16 and CXC and CC chemokine receptors on CD4^+^ T cells is also involved in immune dysfunction of autism (331–334). When it comes to Th cell subsets, there is no doubt that they are essential participants in the pathogenesis of autism. A skewed Th1/Th2 cytokine profile has long been observed in autism patients (335). According to a study on the immunocyte phenotypes of BTBR T^+^Itpr3^tf^/J (BTBR) mice (a classical animal model of autism), BTBR mice exhibited higher levels of Th1 and Th2 cells, along with a lower Th1/Th2 ratio compared to C57BL/6J mice (336). The imbalance between Treg and Th17 cells also plays an important role, with a significant reduction in Treg cells and an increase in Th17 cells reported in ASD patients (337). This kind of imbalance can be modulated by a PARP-1 inhibitor, 5-aminoisoquinolinone, which has potential anti-inflammatory and neuroprotective effects (338).

Th17 cells also exhibit pathogenicity in autism. A number of studies reported an up-regulated IL-6/IL-17A signaling pathway, indicating overactivity of Th17 cells in autism (339–341). It was found in a mouse study that RORγt-dependent T cells such as Th17 cells, which produced IL-17A, played a key role in the induction of autism-like behaviors as well as an atypical cortical phenotype in offspring by MIA (342). Kim et al. found that IL-17A shaped immune-primed phenotypes in murine offspring through MIA-induced alterations of the maternal microbiota, which affected the chromatin accessibility of CD4^+^ T cells (343). This may account for the phenomenon that individuals with neurodevelopmental disorders such as autism are susceptible to intestinal inflammation.

Studies have also been conducted on transcription factor signaling pathways, providing further evidence for Th cell dysfunction in autism. Children with autism displayed dysregulation of Th1, Th2, Treg and Th17 cell-related transcription factor signal passages, characterized by increased RORγt^+^, T-bet^+^ and GATA-3^+^ T cells and Foxp3^+^ Treg cell deficiency, compared to typically developing control children (344). This was reversed by resveratrol in BTBR mice (345). Therapies targeting Th17 and/or Treg pathway have shown encouraging prospects (346–351). VGX-1027 (a strong immunomodulator) and S3I-201 (a selective Stat3 inhibitor) can improve autism symptoms of BTBR mice by extensively regulating Th cell-related cytokines (352, 353). Upregulation of intracellular enzymatic antioxidants of CD4^+^ T cells in autism children was revealed by a study, displaying the potential of oxidants to reduce IL-17A levels (354).

The correlation between Th cells and other mental disorders has also been reported. For example, patients with type 1 bipolar disorder displayed a decline in Treg cell percentage, a more active cytokine production and a bias to Th1 in the Th1/Th2 balance (355). Despite a proinflammatory role in many cases, Th17 cells were reported to make contribution to the maintenance of the integrity of the brain structure and function (356). A study on obsessive compulsive disorder (OCD) found that Th17 cells were able to trigger OCD like behaviors in mice (357). Another study provided supporting evidence, demonstrating higher proportions of Th17 cells and lower proportions of Treg cells in OCD patients (358). Th cell dysregulation is also involved in the development of generalized anxiety disorder (GAD). In GAD patients, Th1 and Th2 cytokines decreased while Th17 cytokines increased (359). Otherwise, CD4^+^ T cells were involved in intermittent explosive disorder-related transcriptional changes (360).

In summary, Th cell abnormalities are widely involved in the pathogenesis of mental disorders (**Table 1**). More in-depth studies are needed to reveal their specific mechanisms for the benefit of treatment methods. Comparatively less studies on the exact mechanisms of Th-mediated psychological pathogenesis were reported, and incongruent observations were also present. Definitive or deterministic conclusions are yet to be discovered. In the future, Th cells and related cytokines may provide effective biomarkers and therapeutic targets for mental disorders.

**Table 1:** Changes of Th cell subsets or their signature cytokine levels in mental disorders.

Mental disorders	Depressive disorder	Schizophrenia	Autism spectrum disorders
Th cell subsets			
Th1 (IFN-γ)	↑ (313)	↓ (317)	↑ (336, 344)
	↓ (304, 308)		
	- (361)		
Th2 (IL-4)	↓ (302)	↑ (317, 328)	↑ (336, 344)
	↑ (303, 304)		
	- (305–307)		
Th1/Th2 ratio	↑ (301, 306, 362)	↓ (317)	↓ (335, 336)
	↓ (304)		
Treg (TGF-β, IL-10)	↓ (295, 309, 312)	↓ (318–320)	↓ (337, 344)
	↑ (311)	↑ (321)	
Th17 (IL-17)	↑ (303, 312–314)	↑ (324–327)	↑ (337, 339–341, 344)
	↓ (315, 316)	↓ (328, 329)	

↑, upregulation; ↓, downregulation; -, no significant change.

## Discussion

The innate functions of Th cells are comparable in neuroinflammatory disorders of different etiologies. The generalized pro-inflammatory roles of Th17 and Th1 cells, as well as the anti-inflammatory role of Th2 and Treg cells are well accepted. But the diseases discussed herein are of different or even contrasting etiologies, and through comparison of Th cell functions in different pathological settings, we hope for more comprehensive understandings of Th cell-mediated immune responses in CNS inflammatory disorders. Indeed, those neuroinflammation-related disorders and their relevance to adaptive immunity were investigated to drastically different degrees. For instance, various in-depth studies correlating Th cells and MS/EAE pathogenesis have been conducted, while roles of Th cells in most mental disorders are merely based on observations and evaluations. As the boundaries of neuroinflammation expand, investigations on the intercorrelated or even shared functions of Th subsets in different disease settings could promote deeper understandings of this complex immune network. In turn, through comparing distinct contributions of Th subsets under different neuroinflammatory conditions, more precise comprehension of pathogenesis, prevention, therapeutics, and prognosis of these diseases may be achieved.

Th1 cells are considered inflammatory and could contribute to disease progression in most cases. However, it was proved that certain schizophrenia patient groups showed Th2- instead of Th1-skewed shift. Also, evidence has suggested that CD4^+^ T cells demonstrate a shift from Th1-phonotype toward others during natural senescence, and this shift could be exaggerated by AD. On the other hand, Th1 function could also be utilized to vaccinate against AD, as Th1 epitopes are pivotal components of established AD vaccines. Th2 and Treg cells deserve their anti-inflammatory descriptions, as excessive or aggressive activation of these two cell types were rarely reported in non-infectious CNS inflammatory disorders. Moreover, the anti-inflammatory nature of Th2 cells is also utilized in preventing severe autoimmune responses during vaccination against AD. However, divergent evidence was also reported, suggesting the deleterious roles of these cells during ageing (178). By comparison, the vicious role of Th17 cells seems applicable. Therefore, Th cells play an interesting yet intricate monopolylogue in the pathogenesis of neuroinflammatory disorders.

Based on prior discussions, it appears evident that a simple dichotomy of “savior or sinner” is far less adequate in defining roles of different Th cell subsets, or their roles in different non-infectious CNS inflammatory disorders. Immunoregulation is inherently intricate and complicated, and when taken together with diversified pathological or physiological factors, the precise manipulation over CNS adaptive immunity in order to achieve better intervention, therapeutics or vaccination seems even more difficult. Major obstacles in the studying on these topics may include: the lack of exact animal models (including several epilepsy and mental disorders), the difficult accessibility to pathological brain samples from patients (such as most mental disorders), the timing of sampling (due to ictal events in epilepsy, relapses and remissions in MS or some mental disorders, and medication choices), the size of clinical samples, as well as the understanding of the so-called inherent etiologies.

An interesting study recently reported that exposure to particulate matter (PM) 10 significantly correlate with expression of CCR6 in CD4^+^ T cells from MS patients (363). Promoted Th17 polarization induced by particulate matter exposure was also reported in the study. Whether PM exposure can elicit similar immunological responses in other neuroinflammatory disorders remains an intriguing question. Moreover, recent studies have also proposed a bunch of novel research strategies associating Th cells and CNS inflammatory disorders, including those concerning gut microbiota changes (364–368), considering sex difference (369–372), as well as utilizing physiomimetic models for *in vitro* interaction studies (373). Conceptually, as Th cells have various intrinsic and constant regulation pathways and functions, studies on Th cells concerning one research field may as well benefit another.

## Author Contributions

WLi devised the outline of the article. WLi, MF and WLu drafted the original manuscript. WLi and MF designed and edited the illustrations. WZ and LM advised on the outline and revised the manuscript. SL provided writing and analyzing instructions. All authors contributed to the article and approved the submitted version.

## Funding

This work is supported by the National Natural Science Foundation of China (82171784, 82171724 and 81970029), the Shaanxi Province Natural Science Foundation (2020JQ082), and College Students’ Innovative Entrepreneurial Training Plan Program, Ministry of Education, China (GJ202110698150 and GJ202110698175).

## Conflict of Interest

The authors declare that the research was conducted in the absence of any commercial or financial relationships that could be construed as a potential conflict of interest.

## Publisher’s Note

All claims expressed in this article are solely those of the authors and do not necessarily represent those of their affiliated organizations, or those of the publisher, the editors and the reviewers. Any product that may be evaluated in this article, or claim that may be made by its manufacturer, is not guaranteed or endorsed by the publisher.
